# Cytoplasmic vacuolization in cell death and survival

**DOI:** 10.18632/oncotarget.10150

**Published:** 2016-06-17

**Authors:** Andrey V. Shubin, Ilya V. Demidyuk, Alexey A. Komissarov, Lola M. Rafieva, Sergey V. Kostrov

**Affiliations:** ^1^ Laboratory of Protein Engineering, Institute of Molecular Genetics, Moscow, Russia; ^2^ Laboratory of Chemical Carcinogenesis, N.N. Blokhin Russian Cancer Research Center, Moscow, Russia; ^3^ Laboratory of Biologically Active Nanostructures, N.F. Gamaleya Institute of Epidemiology and Microbiology, Moscow, Russia

**Keywords:** regulated cell death, vacuolization, microbial toxins, viruses

## Abstract

Cytoplasmic vacuolization (also called cytoplasmic vacuolation) is a well-known morphological phenomenon observed in mammalian cells after exposure to bacterial or viral pathogens as well as to various natural and artificial low-molecular-weight compounds. Vacuolization often accompanies cell death; however, its role in cell death processes remains unclear. This can be attributed to studying vacuolization at the level of morphology for many years. At the same time, new data on the molecular mechanisms of the vacuole formation and structure have become available. In addition, numerous examples of the association between vacuolization and previously unknown cell death types have been reported. Here, we review these data to make a deeper insight into the role of cytoplasmic vacuolization in cell death and survival.

## INTRODUCTION

In the kingdoms of plant, fungi and Protista vacuoles are common intracellular organelles which my occupy a significant proportion of cell volume and serve multiple physical and metabolic functions that are essential to life [1–3]. Unlike plant and fungi cells, most animal cells commonly do not contain vacuoles as regular organelles. At the same time, formation of giant vacuoles in animal cells *in vivo* and in culture occurs as a morphological phenomenon (commonly called cytoplasmic vacuolization or vacuolation) that develops spontaneously or after exposure to bacterial or viral pathogens as well as to various natural and artificial low-molecular-weight compounds [4–6].

Cytoplasmic vacuolization of mammalian cells can be transient or irreversible. Transient vacuolization is observed only during the exposure to an inducer and reversibly affects the cell cycle and migration [7, 8]. Most known inducers of transient vacuolization are weakly basic amine-containing lipophilic compounds. In neutral extracellular fluid, lipophilic bases are uncharged and can be transported through the plasma membrane via passive diffusion or active transport [9, 10]. Within the cell, uncharged lipophilic bases freely diffuse through the organelle membranes. But after entering acidic endosomal-lysosomal organelles and Golgi cisterns, they become positively charged and lose the capacity to diffuse through the organelle membranes back to the cytoplasm. The accumulation of charged forms of weak bases increases the intraorganellar osmotic pressure. The equilibration of osmotic pressure by water diffusion across organelle membranes leads to the formation of the vacuoles [5, 11, 12]. Thus, osmotic effects associated with disturbed ionic balance in the organelles rather than the impact on proteins controlling cellular functions underlie the action of transient vacuolization inducers.

In contrast to transient vacuolization, irreversible vacuolization marks cytopathological conditions leading to cell death, as long as the cytotoxic stimulus is present. In addition to acidic organelles, irreversible vacuolization can affect the endoplasmic reticulum (ER) as well as known non-acidic organelles of the endosomal-lysosomal system and Golgi apparatus. Clearly, the vacuoles are formed in different cellular compartments by different mechanisms. To date, the capacity to induce irreversible cytoplasmic vacuolization has been shown for a variety of natural and synthetic compounds of different chemical structure including medical drugs and industrial pollutants [13–20]. In addition, irreversible vacuolization is observed in cells infected by a multitude of bacterial and viral agents of serious human and animal diseases. In this case, bacterial protein toxins and virus envelope or capsid proteins can serve as vacuolization inducers. It should be noted that the proteins with a vacuolating activity often are the major factors of the cytotoxic effect of pathogens [21–26].

Sometimes irreversible vacuolization accompanies cell death that cannot be attributed to any type recognized to date [27–30]. In contrast, a fraction of inducers of irreversible vacuolization causes known types of caspase-independent cell death including methuosis, paraptosis (and paraptosis-like cell death), oncosis, and necroptosis [31–34]. It is important that these cell death types are typical for tumor cells including apoptosis-resistant cells, which makes their investigation promising for the development of new therapeutic approaches to oncological diseases [35–42].

The above considerations raise the problem about the role of cytoplasmic vacuolization in cell death process. This is the core problem of toxicological, microbiological, and medical studies of vacuolization. The analysis of the data available at the end of the last century suggested that the formation of vacuoles “primarily reflects an adaptive, survival response to a plethora of environmental changes, that also has the potential to lead to a particular and distinctive form of cell death” [4]. New data on the molecular mechanisms of vacuole formation and structure have become available since then, and numerous examples of the association between vacuolization and previously unknown cell death types have been reported. This prompted us to revisit previous suggestions for the role of vacuolization in cell death and survival.

## VACUOLIZATION AND KNOWN CELL DEATH PATHWAYS

### Methuosis

Methuosis is a caspase-independent cell death accompanied by vacuolization of macropinosomes resulting from dysregulation of macropinocytosis [31]. During abnormal macropinocytosis in methuosis, macropinosomes do not fuse with other organelles of the endocytic pathway and do not recirculate to the plasma membrane but rather accumulate in the cytoplasm, fuse with each other, and form vacuoles. The membranes of the vacuoles show no markers of autophagosomes (LC3), early endosomes (Rab5 and EEA1), or endosomes recirculating to the plasma membrane (Rab11). At the same time, the membranes are positive for markers of late endosomes and lysosomes (GTPase Rab7 and membrane glycoprotein Lamp-1). However, in contrast to these organelles, vacuoles contain no hydrolytic enzymes and have non-acidic content (Table 1). Taken together, the properties of vacuoles formed in methuosis allow us to consider them as nonfunctional late endosomes [31, 43].

**Table 1:** Comparison of properties of cytoplasmic vacuoles derived from endosomal-lysosomal organelles

Cell death type or inducer	Markers of organelles								Inhibitors^a^				Demonstrated on cell lines	Ref.
	Lamp 1/2	Rab5	Rab7	Rab9	Rab11	LC3	Extracellular liquid uptake	Acidic pH	3MA	BafA1	Filipin	Microtubule inhibitors		
Methuosis	+	−	+	nd	nd	−	+	−	nd	+	+	nd	U251	[31, 43, 46]
1-methyl-4-phenylpyridinium	+	−	+	nd	nd	+	nd	+	nd	−	nd	nd	SHSY5Y	[28]
Stx2 toxin, *E. coli* (verotoxin-2)	nd	nd	nd	nd	nd	nd	nd	+	nd	+	nd	nd	Vero, CHO	[138, 163]
SubAB toxin, *E. coli*	nd	nd	nd	nd	nd	nd	nd	+	nd	+	nd	nd	Vero	[139, 150]
CARDS toxin, *M. pneumoniae*	+	−	−	+	nd	nd	+	+	nd	+	nd	+	HeLa, Vero, CHO-K1, A549	[137]
VacA toxin, *H. pylori*	+	−	+	−	−	−	+	+	−	+	−	−	HeLa	[23, 142, 180, 189]
Epsilon-toxin, *C. perfringens*	+	−	+	nd	nd	nd	nd	+	−	+	nd	+	MDCK	[25]
Cytolysin, *V. cholerae*	+	nd	+	−	−	+	+	+	+	−	nd	nd	Vero, MDCK, HeLa, BHK, A431, T84	[205, 206]
Binary toxin, *B. sphaericus*	+	−	+	nd	nd	+	nd	+	nd	nd	nd	nd	MDCK	[144]
3C protease, Hepatitis A virus	+	+	+	+	+	+	+	−	−	+	−	−	A549, Calu-1	[30]

a 3MA - 3-methyladenine; BafA1 - Bafilomycin A1

nd - no data

The first data on the molecular mechanisms of methuosis were obtained in studies of mutation profiles in glioblastoma and gastric cancer cells. It was shown that Ras-activating mutations typical of malignant tumors of different origin are extremely rare in glioblastoma and gastric cancer cells [35, 44]. The clue to this was found in experiments demonstrating that the expression of oncogenic HRAS and KRAS (coding for constitutively active GTPases H-RASG12V and K-RAS4BG12V, respectively) in gastric carcinoma and glioblastoma cells induces their death by methuosis [31, 45]. A similar effect was demonstrated later for osteosarcoma and HEK293 cells [46].

**Figure 1:**
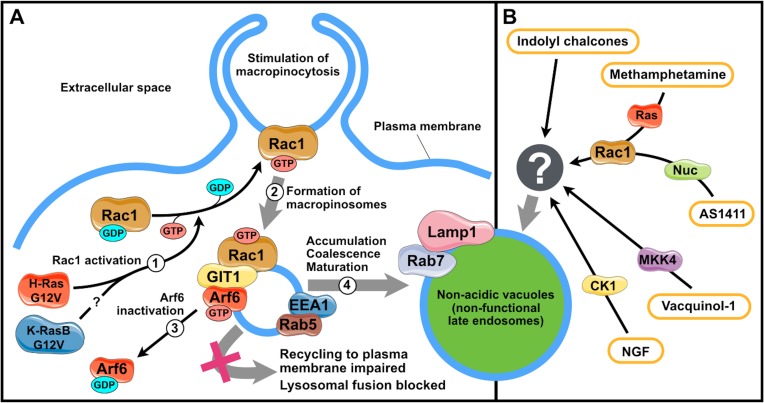
Mechanisms of vacuolization triggered by methuosis inducers **A.** Stages of vacuole formation induced by Ras oncogenes. 1, H-RasG12V and (presumably) K-RasBG12V activate GTPase Rac1; 2, activated Rac1 stimulates macropinocytosis; 3, active Rac1 associates with GIT1 to inactivate Arf6, which blocks the recycling of macropinosomes to the plasma membrane; 4, accumulated macropinosomes acquire some properties of late endosomes and fuse to form vacuoles. **B.** Vacuole formation triggered by other methuosis inducers. CK1, casein kinase 1; MKK4, mitogen-activated protein kinase kinase 4; Nuc, nucleolin; GIT1, G protein-coupled receptor kinase interacting ArfGAP 1; Arf6, ADP-ribosylation factor 6; EEA1, early endosome antigen 1; NGF, nerve growth factor. The figure was produced using Servier Medical Art (http://www.servier.com).

Ras-induced methuosis is accompanied by activation of caspases; however, their suppression has no effect on cell survival [31]. Methuosis process does not depend on the activity of kinase PI3K class I, GTPase RhoA, and GTP-binding proteins RalA and CDC42, downstream effectors of Ras oncogenes. In addition, methuosis is unrelated to Ras-Raf-MEK-ERK1/2 signaling [45, 46]. At the same time, H-RASG12V can activate GTPase Rac1, which controls the formation of macropinosomes at the stage when lamellipodia at the plasma membrane emerge [46, 47] (Figure 1A). The constitutive activation of Rac1 *per se* is sufficient for the formation of vacuolated macropinosomes [48]. In addition to macropinocytosis induction, active Rac1 interacts with the adapter protein GIT1, a suppressor of the GTP-binding protein Arf6 controlling pinosome recycling to the plasma membrane; this leads to the accumulation of numerous macropinosomes, their abnormal fusion, and vacuole formation. Apparently, the same mechanism takes place in methuosis induction by constitutively active K-RAS4BG12V [46].

In the last five years, a number of factors inducing death of cells of different origin accompanied by macropinosome vacuolization have been discovered. Methamphetamine induces methuosis in SH-SY5Y neuroblastoma cells [49, 50]; indolyl chalcones (1, 3-diphenyl-2-propen-1-one derivatives), in U251 glioblastoma, LN229 glioma, U2OS osteosarcoma, MCF7 mammary tumor, SW480 rectal cancer, and PANC-1 pancreatic cancer cells [43, 51]; vacquinols (quinin derivatives), in several glioblastoma cell lines [52]; DNA aptamer AS1411, in DU145 prostatic cancer cells [53]; and nerve growth factor, in Daoy medulloblastoma cells [54]. Only limited data is currently available on the mechanisms underlying the effect of these substances. Still, these data suggest that their action differs from that of constitutively active Ras. For instance, indolyl chalcones induce no changes in Rac1 and Arf6 activities; the effect of methamphetamine depends on Rac1 and Arf6 but no caspases are activated [49, 50]; macropinocytosis induction by vacquinols requires active kinase MKK4 [52] (for a detailed review see [42]); the effect of nerve growth factor depends on casein kinase 1 activity [54]; and AS1411-induced macropinocytosis depends on EGFR and Rac1, and is negatively regulated by nucleolin, a protein ligand of AS1411 [53] (Figure 1B).

To date, methuosis has been demonstrated practically in malignant cells only. This fact allows to consider inducers of methuosis as promising antitumor therapeutic agents [42]. The factors of cancer cells susceptibility to methuosis remain unknown. Presumably, it results from modified regulations of endocytosis and endosomal traffic observed in malignant transformation.

It is also obscure what causes cell death in methuosis. The initial assumption was that the accumulation of vacuoles is inconsistent with normal cell functioning and causes cell death. Indeed, a correlation between cellular disintegration and increase in the number and size of vacuolated macropinosomes is observed in Ras-induced methuosis [31]. This assumption is also supported by the fact that the exposure of SH-SY5Y neuroblastoma cells to a macropinocytosis inhibitor prevented not only vacuolization but also methamphetamine-induced cell death [49]. However, certain chalcone derivatives proved to induce pronounced macropinosome vacuolization without cell death [20], which indicates that vacuolization *per se* is not a factor of cell death at least for certain methuosis inducers.

### Paraptosis and paraptosis-like cell death

Paraptosis is a type of cell death with the vacuolization of ER components and swelling of mitochondria. Paraptosis was first observed after the overexpression of the IGF1R receptor in primary mouse fibroblasts and several types of human cancer cells [37]. Paraptosis process requires that the transcription and translation systems are functioning [32], and its mediators include caspase-9 (but not other caspases), multifunctional protein prohibitin, as well as kinases of the MAPK/ERK and JNK/SAPK pathways [55]. Paraptosis inhibitors known to date include the multifunctional adapter protein AIP1/Alix and phosphatidylethanolamine-binding protein 1 (PEBP-1). The effect of AIP1/Alix relies on the inhibition of IGF1R phosphorylation, which blocks the activation of the MAPK/ERK and JNK/SAPK signaling pathways [32, 55–57]. The effect of PEBP-1 is likely due to its capacity to block the activation of JNK/SAPK signaling [55]. It should be noted that PEBP-1 can also block the activation of MAPK/ERK signaling through the interaction with Raf-1 [58]. However, PEBP-1 has no effect on the activation of this signaling pathway in paraptosis. This can be attributed to the MAPK/ERK activation downstream of Raf-1 or by another kinase in paraptosis [55].

Current data indicate that cell death with ER vacuolization and mitochondrial swelling typical for IGF1R-induced paraptosis can be initiated by a number of other stimuli including oxidative stress [59–61], overexpression of TAJ/TROY receptors [62], epidermal growth factor (EGF) [63, 64], glucocorticoids [65], thiol reactive cyclopentenone prostaglandin 15d-PGJ2 [66] as well as various xenobiotics [16, 67–74]. At the same time, cell death triggered by these inducers relies on the mechanisms different from that of IGF1R-induced paraptosis (Table 2). For instance, no chromatin fragmentation is observed during paraptosis, while the death of *Dictyostelium discoideum* cells in oxidative stress or rat pituitary cell death induced by EGF is accompanied by nuclear DNA degradation mediated by L-DNAse-II or apoptosis-inducing factor, AIF [60, 63, 64]. In some cases, cell death with paraptotic features can be independent of the transcription and translation systems [75], activation of the MAPK/ERK [70, 74] and JNK/SAPK [66, 75] pathways or can be mediated by p38 MAP kinase activity [69, 71, 74, 76]. Cell death and ER vacuolization induced by 15d-PGJ2 and manumycin A is accompanied by a sharp increase in production and processing levels of LC3, which is one of the main components of autophagy machinery. However, vacuolization and cell death depend only on the presence of the LC3, but not on autophagy [29, 66]. Thus, the morphology typical of paraptosis is observed during several cell death types with different biochemical mechanisms, which are commonly called “paraptosis-like cell death” (PLCD).

**Table 2:** Action of inducers of paraptosis and paraptosis-like cell death

Inducer^a^	Activation of			Inhibition by^b^				Demonstrated on cell lines	Ref.
	MAPK/ERK	JNK/SAPK	p38MAPK	AIP1/Alix	Bcl-XL	Bcl-2	ActD/CHX		
IGF1R	+	+	−	+	−	nd	+	HEK293T	[32, 55]
EGF	−	−	−	+	nd	+	nd	GH4C1	[63, 64]
Corticosteroids	nd	+^c^	nd	+	nd	nd	nd	ARPE-19, rat retinal pigment epithelial cells	[65]
Yessotoxin	nd	+	+	nd	nd	nd	nd	BC3H1	[16, 69]
1-Nitropyrene	+	+	+	nd	nd	nd	+	Hepa1c1c7	[71]
Taxol	−	−	−	nd	nd	−	−	ASTC-a-1, HeLa, U87	[75]
WIN55,212-2	−	nd	nd	nd	nd	nd	+	Granta519, Rec1, JeKo, JVM2, primary mantle lymphoma	[70]
15d-PDJ2	+	+^d^	+^d^	nd	nd	nd	+	HCT116, DU145, MDAMB231	[29]

a 15d-PDJ2 – 15-deoxy-Δ12,14-prostaglandin J2; WIN55,212-2 – (R)-(+)-[2,3-Dihydro-5-methyl-3-(4-morpholinylmethyl)pyrrolo[1,2,3-de]-1,4-benzoxazin-6-yl]-1-napthalenylmethanone

b ActD – actinomycin D; CHX – cycloheximide

c Phosphorylation of JNK-2 but not JNK-1

d Marginal activation, inhibitors have no effect

nd, no data

**Figure 2:**
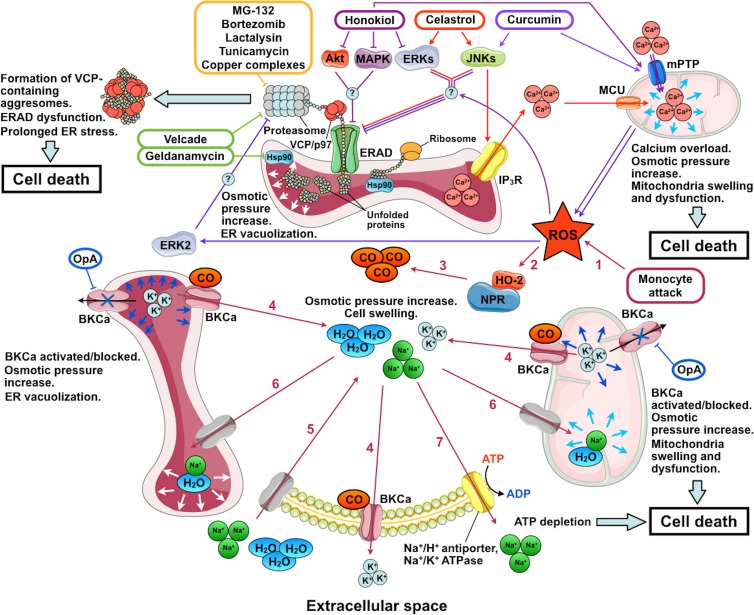
Mechanisms of action of inducers of paraptosis and paraptosis-like cell death The names of the inducers of paraptosis and paraptosis-like cell death (PLCD) are given in colored frames; their pathways are marked by arrows of the corresponding color. Stages of PLCD induced by activation of big conductance calcium-activated potassium channels (BKCa) by reactive oxygen species (ROS) are indicated by numbers: 1, ROS enter the cytoplasm; 2, ROS activates heme-oxygenase 2 (HO-2) and NADPH-P450 reductase (NPR); 3, carbon monoxide (CO) is produced by HO-2 and NPR; 4, BKCa activation by CO leads to K^+^ release from the ER and mitochondria to the cytoplasm and from the cytoplasm to the extracellular space; 5, low intracellular K^+^ concentration is compensated by the entry of Na^+^ accompanied by water into the cell; 6, low K^+^ concentration in the ER and mitochondria is compensated by the entry of Na^+^ and water, which leads to mitochondrial swelling and ER vacuolization; 7, intracellular Na^+^ concentration decreases through the activation of Na^+^/H^+^-antiporters and Na^+^/K^+^ ATPase. Under conditions of mitochondrial dysfunction induced by ROS and BKCa opening, the activity of ATP-dependent Na^+^ transporters depletes the ATP pool and causes cell death. OpA, ophiobolin A; IP_3_R, inositol trisphosphate receptor; ERAD, endoplasmic reticulum-associated protein degradation; Hsp90, heat shock protein 90; VCP/p97, valosin-containing protein; mPTP, mitochondrial permeability transition pore; and MCU, mitochondrial calcium uniporter. The figure was produced using Servier Medical Art (http://www.servier.com).

The mechanism of ER vacuolization in IGF1R-induced paraptosis currently remains unidentified. At the same time, the mechanisms of ER vacuolization during certain PLCD types are largely clear; it is dysfunction of either endoplasmic reticulum-associated protein degradation (ERAD) or ER-localized big conductance calcium-activated potassium channels (BKCa) (Figure 2). Considering large volumes of data accumulated on these cases of vacuolization, they are covered in detail below.

#### ER vacuolization due to dysfunctional ERAD

Endoplasmic reticulum-associated protein degradation provides for the identification of folding-defective proteins in the ER, their translocation to the cytoplasm, and degradation by the ubiquitin-proteasome system (reviewed in [77, 78]). ER vacuolization due to dysfunctional ERAD has been most extensively studied for the combined inhibition of HSP90 by geldanamycin and proteasomes by Velcade (bortezomib) [36, 79] (Figure 2). HSP90 inhibition leads to the accumulation of misfolded proteins in the ER. These proteins are recognized by the ERAD system and transferred to the cytoplasm by retrotranslocation dependent on valosin-containing protein (VCP). At the same time, proteasome inhibition by Velcade leads to the formation of insoluble perinuclear aggresomes composed of VCP complexes with undegraded polyubiquitinated proteins. As polyubiquitinilated proteins are accumulated in the cytoplasm, all VCP is transferred from the ER membranes and cytoplasm to aggresomes. This suppresses ERAD activity, which results in the accumulation of considerable quantities of misfolded proteins in the ER and leads to ER stress. Long-term unresolved ER stress causes cell death. The vacuolization of ER components after the combined exposure to geldanamycin and Velcade seem to be due to the osmotic pressure increase resulting from high quantities of misfolded proteins in the ER. The compensation of osmotic pressure by water diffusion into the ER leads to the formation of vacuoles. The overexpression of dominant-negative VCP, an ERAD inhibitor, also leads to ER vacuolization, which confirms the relationship between ERAD and vacuolization. At the same time, cell exposure to inhibitors of translation and transcription that prevented the protein inflow to the ER completely suppressed the vacuolization [36, 79, 80].

In addition to geldanamycin and Velcade, PLCD and ER vacuolization can be triggered by inhibitor of HSP70 VER155008 [81], other ERAD inhibitors (e.g., thioxotriazole or phosphine copper complexes [15, 82], gold complexes [83]), proteasome inhibitors MG-132, lactalysin, and tunicamycin [79, 80], as well as a number of compounds not directly interacting with ERAD components. The latter can be conventionally divided into two groups. The first group includes celastrol from *Tripterygium wilfordii* [76], capsaicin from *Capsicum* plants [84], hesperidin from *Citrus* plants [85], gypenoside L from *Gynostemma pentaphyllum* [86], 1-nitropyrene [71], and cannabinoid receptor antagonists from *Cannabis sativa* as well as their synthetic derivatives [70]. The effect of these substances is mediated by the activation of one or several signaling pathways (MAPK/ERK, JNK/SAPK, and p38MAPK). To date, no data are available on the mechanisms underlying the effect of inducers of this group apart from celastrol, hesperidin and gypenoside L.

The main event in celastrol action critical for cell death is the induced release of Ca^2+^ ions through inositol triphosphate receptor (IP3R) from the ER to the cytoplasm and the subsequent Ca^2+^ entry into the mitochondria via the mitochondrial calcium uniporter (MCU). In addition, celastrol was proposed to inhibit proteasomes and impair protein folding in the ER (possibly by modulating the ERK and JNK signaling pathways) [87]. Mitochondrial overload with Ca^2+^ leads to their dysfunction and formation of reactive oxygen species (ROS), which further inhibit proteasomes and affect protein folding in the ER. Proteasome inhibition prevents the degradation of IP3R and MCU, which reinforces Ca^2+^-mediated effects of celastrol and leads to ERAD dysfunction and ER vacuolization. Ultimately, these events cause general stress and death of the cell [87] (Figure 2). Apparently, a similar situation is observed in PLCD induced by gypenoside L and hesperidin. In case of gypenoside L the increase of the intracellular ROS levels is the initial event, which, in turn, triggered protein ubiquitination and unfolded protein response (UPR), resulting in Ca^2+^ release from IP3R-operated ER stores and, finally, in cytoplasmic vacuolation and cell death [86] In case of hesperidin, however, ryanodine receptors mediate Ca^2+^ release from the ER in addition to IP3R [85].

The second group of vacuolization inducers not directly interacting with ERAD components includes curcumin from *Curcuma longa*, honokiol from *Magnolia grandiflora* seed extract, as well as ginsenoside Rh2 and protopanaxodiol (PPD) from *Panax ginseng* root. The action of these compounds depends not only on the triggering of the MAPK/ERK and/or JNK/SAPK pathways but also on the presence of ROS in the cell.

Curcumin and honokiol selectively induce PLCD in breast and digestive tract cancer cells. Accordingly, curcumin and honokiol as well as their derivatives are considered as potential anticancer agents [38, 88–97]. Curcumin is known to demonstrate both antioxidant and ROS-generating activities [98, 99]. When intracellular concentration is low, curcumin activates natural antioxidation mechanisms in cells, allowing efficient scavenging of free radicals and protection from lipid peroxidation and DNA damage [100, 101]. In contrast, when the concentration is high, curcumin seems to disturb antioxidant capacity of cells and exert cytotoxic effect [102–106]. As an PLCD inductor, curcumin increases the mitochondrial level of Ca^2+^, possibly through opening the mitochondrial permeability transition pore, mPTP [107]. This gives rise to considerable ROS quantities, which activate ERK2. Irrespective of the induction of ROS production, curcumin activates JNK and downregulates the expression of the paraptosis inhibitor Alix/AIP1. The activation of JNK and ERK2 inhibits proteasomes by an unknown mechanism, which leads to ERAD dysfunction as well as ER stress and vacuolization [103, 108] (Figure 2). Apart from that, inhibition of proteasomes prevents the activation of the transcription factor NF-κB, which makes cancer cells sensitive to cell death inducers [109, 110]. The effect of honokiol is similar to that of curcumin: it was also shown to promote mPTP opening and ROS accumulation [111]; although honokiol inhibits rather than induces MAPK/ERK signaling [112, 113] (Figure 2). Similar to IGF1R, curcumin and honokiol activities are suppressed by Alix/AIP1 overexpression, inhibitors of transcription/translation, antioxidants, and inhibitors of the JNK/SAPK and (for curcumin) MAPK/ERK pathways [94, 103, 114].

PLCD induction by ginsenoside Rh2 and PPD has been shown in colorectal cancer cells. These drugs also trigger MAPK/ERK signaling and lead to the accumulation of ROS [115, 116]. However, in contrast to curcumin and honokiol whose action is blocked by antioxidants, the cytotoxic effect of Rh2 and PPD increases in the presence of antioxidants. Thus, ROS are required for PLCD in the case of curcumin and honokiol; while in the case of Rh2 and PPD, ROS inhibit cell death. It remains unclear what underlies the opposite effects of ROS. The protective function in the case of Rh2 and PPD can be due to specific regulation of NF-κB signaling in colorectal cancer cells [114, 117].

#### ER vacuolization due to dysfunctional BKCa

Big conductance calcium-activated potassium channels (BKCa) are localized to the membranes of the ER and mitochondria as well as to the plasma membrane of the cell; they are involved in the regulation of many intracellular processes (reviewed in [118]). The relationship between ER vacuolization and BKCa activity was first demonstrated in the studies of the mechanisms of cytotoxic effect of human monocytes on glioma cells [39, 119] (Figure 2). Excessive ROS introduced to glioma cells by monocytes stimulate NADPH-P450 reductase (NPR) and hemoxygenase-2 (HO) to produce carbon monoxide, a BKCa activator. BKCa opening releases K^+^ from mitochondria to cytoplasm and from cytoplasm to extracellular fluid. K^+^ release from the cell is compensated by the influx of extracellular Na^+^. Water accompanies Na^+^ to the cell, ER, and mitochondria. This increases the cell volume and leads to mitochondrial swelling and ER vacuolization. Cellular systems of ion homeostasis tend to decrease Na^+^ concentration through the activation of ATP-dependent Na^+^/H^+^ antiporters and Na^+^/K^+^ ATPase. In conditions of mitochondrial dysfunction induced by BKCa opening and ROS, the activity of ATP-dependent Na^+^ transporters rapidly depletes the ATP pool and causes cell death [39].

BKCa inhibition can also induce ER vacuolization. Such induction is observed after the exposure of tumor cells to ophiobolin A (OP-A), a BKCa inhibiting metabolite with an anticancer activity from the fungus *Bipolaris* sp. [40, 120, 121]. BKCa inhibition was proposed to give rise to excessive K^+^ in the ER and mitochondria. The equilibration of K^+^ concentration mediated by the osmotic effects leads to the formation of vacuoles (Figure 2). In addition, impaired K^+^ homeostasis leads to the activation of calcium channels in the plasma membrane and Ca^2+^ influx into the cell. Presumably, a long-term increase in Ca^2+^ concentration in the cytoplasm is the cause of cell death [40]. Apart from that, the cytotoxic effect of OP-A on tumor cells is attributed to its capacity to suppress the main oncogenic pathways: PI3K/mTOR, Ras/Raf/ERK, and CDK/RB [120].

The following conclusions concerning the significance of vacuolization in PLCD process can be drawn from the data available on the mechanisms of ER vacuolization. Under conditions of affected BKCa, cell death is not directly related to ER functioning, and vacuolization most likely has no effect on the cell death process. Conversely, under conditions of affected ERAD, cells die from long-term ER dysfunction. However, the increased ER volume provided by vacuolization can decrease the concentration of misfolded proteins in the ER and facilitate their utilization [36]. Thus, ER vacuolization in ER dysfunction can be considered as an adaptive cellular response aimed at cell survival under stress.

### Necroptosis and oncosis

Necroptosis and oncosis represent necrosis-like cell death types demonstrating mitochondrial swelling and vacuolization of ER and Golgi components. Considering that these cell death types are primarily typical for malignant cells, the mechanisms of oncosis and necroptosis processes are actively studied [41].

It has been established that necroptosis is a type of programmed necrosis developed after the activation of death receptors when caspases and the inhibitor of apoptosis protein (IAP) are deficient. Receptor interacting protein (RIP) kinases 1 and 3 as well as pseudokinase MLKL are the main regulators of necroptosis [122–125] (also reviewed in [126]). Despite active studies of necroptosis, no data are available on the mechanisms underlying the accompanying vacuolization.

For a long period of time, oncosis was considered as a passive accidental form of cell death developed in ischemia, mechanical tissue injury, and intoxication [127–131]. However, recent data indicate that cell death by oncosis can be initiated by the activation of cell surface PORIMIN (pre-oncosis receptor induced membrane injury) receptors, a modest increase in the expression of uncoupling protein 2 (UCP2), and overexpression of chromatin modifying protein 6 (CHMP6). Oncosis process depends on perforin-1 and caspase-1, while the transcription factor NFκB protects cells against oncosis [33, 132]. These data allow us to consider oncosis as a regulated cell death.

The mechanisms of oncosis are poorly understood. It is known that the key event in oncosis is the early opening of the mPTP. This collapses the mitochondrial transmembrane potential and stops ATP synthesis. In the absence of ATP, ATP-dependent ion pumps in the plasma and organellar membranes cannot function. This irreversibly affects ion transport through cell membranes, which modifies the ionic composition of cytoplasm and intraorganellar fluids. Altogether, these events culminate in cell death, while the emerging osmotic effects apparently induce vacuolization (reviewed in [33]). Thus, vacuolization appears not to be the cause of cell death in oncosis.

## VACUOLIZATION AND UNKNOWN CELL DEATH PATHWAYS

### Low molecular weight inductors

In addition to the above cases, there are multiple examples of cell death accompanying by vacuolization that cannot be attributed to any type recognized to date.

Cell death of unrecognized types may be induced by certain low-molecular weight compounds. One of such examples is action of 1-methyl-4-phenylpyridinium (MPP^+^) environmental toxin on SHSY5Y cells resulting in sustainable activation of ERK, arrest of autolysosome maturation and consequent formation of acidic autolysosomal vacuoles [28]. The vacuolization can be ameliorated by interleukine-6, which promotes maturation of autolysosomes in MPP^+^-treated cells. Although the exact cause of MPP^+^-induced vacuolization is unknown, prolonged ERK activation is discussed as the main reason of blockage of final steps of autophagy and formation of vacuolated defective autolysosomes, as it was shown in several cases of vacuolization not associated with cell death [133, 134].

Another example is an atypical cell death of HEK293 cells, hippocampal, striatal, and cortical neurons induced by interaction of neuropeptide substance P (SP) with neurokinin-1 receptor (Nk_1_R), and accompanied by autophagosomal vacuolization [27, 135]. SP-inducedcell death and vacuolization depend on autophagy and require functioning of core autophagy machinery proteins PI3K-III, Beclin-1 and Atg7. Activation of Raf-1, MEK2 and ERK2 leading to phosphorylation of nuclear receptor NR4A1 is essential for SP-induced/Nk_1_R-mediated cell death [27, 136]. It is of note that phosphorylation of NR4A1 through activation of Ras/MEK/ERK signaling pathways is also demonstrated for IGF1R-induced HEK293 cell death, which is also autophagy-dependent [27]. Therefore, one may suggest that NR4A1 is a modulator of several types of cell death associated with autophagy.

For all the described cases there are too few data to make any suggestions on a role of vacuolization in the cell death progress.

### Bacterial infections

Cytoplasmic vacuolization is observed after the exposure of human and animal cells to many bacterial pathogens. The mechanisms of cell death and vacuolization remain unclear for most of them. At the same time, in all studied cases vacuolization is induced by secreted protein toxins of bacteria. This section reviews the mechanisms of actions for known bacterial vacuolization-inducing toxins, which fall into several structural families and have ADP-ribosyltransferase [137], N-glycosidase [138], proteolytic [139–141] or ion channel-forming activities [142–146].

### Toxins with enzyme activities

#### Escherichia coli toxins Stx2 and SubAB

Secreted toxins Stx2 and SubAB are virulence factors of pathogenic *E. coli* serotypes that cause diarrheal diseases complicated by hemolytic-uremic syndrome, acute renal failure, or central nervous system disorders [21, 138, 147, 148]. Stx2 and SubAB belong to the AB(5) family; i.e., are protein complexes composed of a single subunit A (SubA) and five subunits B (SubB). SubA has a catalytic activity and is responsible for the cytotoxic effect. SubB bind to receptors on the surface of the cell membrane and provide for toxin entry into the cell [149].

During infection, Stx2 and SubAB bind to the surface of endothelial cells in the digestive tract and renal glomeruli, induce endocytosis, and enter early endosomes. In endosomes, they are transported to the Golgi and then to the ER [138, 150]. Both toxins induce vacuolization and cell death [151, 152]; however, their A-subunits have different catalytic activities and B-subunits utilize different receptors to enter the cell. Stx2 SubA has N-glycosidase activity towards 28S ribosomal RNA, and globotriaosylceramide is the receptor of SubB. In the case of SubAB, SubA has proteolytic activity towards chaperone BiP (also known as HSPA5), which assists in the folding of cellular proteins in the ER [147, 153]. Sialylated proteins with terminal monosaccharide N-glycolylneuraminic acid serve as receptors for the SubB [154]. The highest affinity to the SubB was shown for the α2β1 integrin receptor, L1 cell adhesion molecule, and hepatocyte growth factor receptor [139].

Vacuoles induced by SubAB and Stx2 have acidic content, and their formation requires active vacuolar ATPase (V-ATPase) (Table 1). This indicates the origin of vacuoles from acidic endosomal-lysosomal organelles. At the same time, neither SubAB nor Stx2 enter acidic endosomal-lysosomal organelles and are not detected in the vacuoles [150]. Thus, SubAB and Stx2 induce vacuolization of the organelles where they are not localized.

In the case of SubAB, only SubB has the vacuolating activity. The underlying mechanism has not been completely described; however, the formation of vacuoles requires the interaction between SubB and α2β1 integrin receptors [155]. It was reported that the β1 subunit of integrin receptors can interact with the 16 kDa subunit of the transmembrane domain of V-ATPase, which is critical for the assembly and functioning of the entire V-ATPase complex [156, 157]. The requirement for active V-ATPase for vacuole formation prompts the suggestion that the vacuolating effect of SubAB relies on the V-ATPase activation by the interaction between SubB of the toxin and α2β1 integrin receptors [155]. It should be noted that the effect of SubAB is not the only case of involvement of integrin receptors in vacuolization process. The activation of α2β1 and other β1 subunit-containing receptors is required for vacuolization in endothelial cells during capillary formation in mammalian tissues [158–160]. In this case, vacuolization depends on pinocytosis-regulating GTPases Rac1 and Cdc42, and the vacuoles form after pinosomes fuse with each other [161]. To date, it remains unclear if this mechanism is realized in SubAB-induced vacuole formation.

The relationship between the vacuolating activity of SubB and SubAB-induced cell death is obscure. Experiments on Vero cells demonstrated that cells with SubB-induced vacuolization remain attached to the substrate over a long period of time but almost cease to grow and eventually die. Thus, SubB causes cell death; however, its cytotoxic effect develops much slower compared to the entire toxin, which causes cell death by mitochondria-mediated caspase-dependent apoptosis [21]. At the same time, experiments on HeLa cells demonstrated that blocked interaction between the toxin and α2β1 integrin receptors substantially decreased the Bax/Bak activation, cytochrome c release, and cell death rate. Nonetheless, the quantity of BiP protein digested by the toxin is not decreased. These data point to the significance of interaction between SubB and α2β1 integrin receptors in cytotoxic effect of SubAB [162].

The mechanism of vacuolating activity of Stx2 also remains largely unclear. Stx2 localization to Golgi and ER is not required for vacuolization process since the vacuoles are formed when Stx2 resides in transport endosomes [163]. It is of note that the Shiga-like toxin 1 (Stx1, a structural homologue of Stx2, which also cleaves 28S rRNA) has no vacuolating activity. Similar to Stx2, Stx1 is transported to the ER via Golgi [164] but, in contrast to Stx2, within transferrin receptor-negative endosomes. This suggests that the localization of Stx2 in transferrin receptor-positive endosomes allows the toxin to trigger an unknown signaling resulting in vacuole formation [138].

Although the mechanisms of vacuolating activity of SubAB and Stx2 as well as the relationship between vacuolization and cell death remain unclear, the data available on the cell death induction without vacuolization indicate that these events are uncoupled. SubAB toxin causes cell death in concentrations from 1 μg/ml, while its vacuolating effect is observed at concentrations from 10 μg/ml [21]. A similar effect was demonstrated for Stx2. In addition, Stx2 can induce cell death in certain lines (e.g., HeLa and Hep2) without vacuolization [138]. This suggests that vacuolization is not the primary cause or prerequisite for Stx2- and SubAB-induced cell death.

#### Mycoplasma pneumoniae CARDS toxin

The vacuolating CARDS toxin (CARDStx) from *Mycoplasma pneumoniae* was discovered relatively recently [165]. CARDStx administration to the murine respiratory tract induces vacuolization and cell death in the bronchial epithelium as well as inflammatory response with signs typical of *M. pneumoniae* infection [24, 165–167]. CARDStx has ADP-ribosyltransferase activity and enters the cell by clathrin-mediated endocytosis using transmembrane annexin A2 as the receptor [168, 169]. Inhibition of the CARDStx catalytic activity suppresses, but not completely, its vacuolating activity [165]. At the same time, expression of the C-terminal region of the toxin alone reproduces its vacuolating effect without inducing cell death [170]. Thus, the vacuolating effect of CARDStx is not due to its catalytic activity or to its cytotoxicity.

The membranes of vacuoles induced by CARDStx are associated with glycoproteins LAMP-1 and LAMP-2, markers of late endosomes and lysosomes, as well as with GTPase Rab9, a marker of late endosomes recirculating to the Golgi. In this case, vacuole formation depends on the GTPase activity of Rab9 and is blocked by the V-ATPase inhibitor bafilomycin A1 or by the ionophore monensin facilitating proton transfer from the acidic organelles to the cytoplasm (Table 1). Overall, these results suggest that CARDStx induces vacuolization of late endosomes recirculating to the Golgi [142, 171].

In conclusion, the available data indicate that bacterial toxins SubAB, Stx2, and CARDStx induce vacuolization of several types of endosomal-lysosomal organelles using individual mechanisms of action independent of their catalytic activity. At the same time, vacuolization in all considered cases is not the primary cause of cell death.

#### Pore-forming toxins

##### Helicobacter pylori vacuolating toxin A

The vacuolating toxin A (VacA) is the best-studied vacuolating pore-forming toxin (PFT). It is the major virulence factor of *Helicobacter pylori*, a bacterium colonizing the gastric mucosa and linked to an increased risk of gastric ulcer, adenocarcinoma, and lymphoma [172].

VacA interacts with many components on cell plasma membrane and exerts multiple effects on susceptible cells [173]. This situation complicates the study of factors required for uptake of the toxin into the cell and development of vacuolization. However, it was found that, depending on cell type, low-density lipoprotein receptor-related protein 1 (LRP1) [174], receptor-like protein tyrosine phosphatases beta (RPTPβ) [175] and alpha (RPTPα) [176], and sphingomyelin [177] are the main cell receptors mediating vacuolating activity of the toxin. Upon binding LRP1, VacA forms anion-selective channels in plasma membrane and is internalized into LRP1-enriched endosomal compartment. After internalization, VacA decreases intracellular glutathione levels, causing accumulation of ROS and initiation of ROS-dependent autophagy [178]. These events results in formation of VacA- and LRP1-enriched autophagosomes and autolysosomes [179]. After binding to RPTPβ on plasma membrane, VacA associates with glycosylphosphatidylinositol-rich lipid rafts, forms anion-seletive channels and activates Rac1, which initiates clathrin-independent endocytosis and provides internalization of the toxin into early endosomes. From the early endosomes VacA is transported to acidic endosomal organelles - late endosomes and lysosomes [175, 180–183]. Although there is no direct experimental evidence, the same mechanism is proposed for RPTPα receptor.

When localized in membranes of late endosomes and lysosomes, VacA channels transport chloride anions (and, with a lower efficiency, Br^−^, I^−^, SCN^−^, pyruvate anions [184]) into the lumen of the organelles, thus decreasing the membrane electrochemical potential [23]. This raises the activity of V-ATPase, which consequently increases the concentration of H^+^ within the organelles [185]. Acidification of the interior favors the accumulation of protonated forms of amino-containing weak bases. (Ammonia synthesized by bacteria to neutralize acidic gastric juice acts as a weak base in *H. pylori* infection [186, 187].) Accumulated amino-containing weak bases increase the osmotic pressure within the organelles. The compensation of osmotic pressure by water diffusion from the cytoplasm leads to vacuolization of endosomes and lysosomes [184] (Table 1). Blocking the anion-selective channel activity of VacA completely suppresses its vacuolating effect [23, 188].

The VacA-induced vacuoles are many times larger than the endosomal-lysosomal organelles. This raised the question about the source of the membrane material to form vacuoles. Currently, two hypotheses have been proposed. According to the first one, vacuoles form by fusing late endosomes and lysosomes with VacA-containing membranes. This hypothesis is supported by the dependence of vacuolization on the activity of proteins meditating the fusion of endosomal-lysosomal organelles: GTPase Rab7 [189], syntaxin 7 [190], VAMP7 [191], and dynamin [192]. According to the second one, vacuoles are formed from individual endosomes and lysosomes enlarged owing to the membrane material within organelles, which was confirmed by direct observation of isolated organelles in the presence of VacA [184, 193]. Since experimental data confirm both hypotheses, parallel utilization of both mechanisms can be assumed. It is of note that VacA-containing autophagosomes and autolysosomes resulted from LRP1-dependent VacA internalization comprise a separate population of organelles and do not fuse to the vacuoles [179].

Although vacuolization notably affects the homeostasis of endosomal-lysosomal organelles, it is not the cause of VacA-induced cell death [194–196]. VacA induces apoptosis through its action on mitochondria, autophagy and connexin 43 (Cx43) turnover [174, 179, 194, 196–199]. VacA effect on mitochondria relies on the formation of anion-selective channels in the inner mitochondrial membrane, whose activity decreases the transmembrane potential [200]. In addition, VacA induces mitochondrial recruitment of Drp1 protein, which leads to the fragmentation of the mitochondrial network [198]. Activation of autophagy, as it was described above, is provoked by VacA-induced ROS. At the same time, ROS activate Rac1 followed by ERK phosphorylation and increase of Cx43 internalization from plasma membrane. Cx43 enters endosome and pre-autophagy compartments, colocolizes with VacA and acquires resistance to autophagic degradation. These events lead to accumulation of undegraded Cx43 in autophagic organelles. Ultimately, autophagy activation along with inhibited Cx43 turnover is proposed to cause cell death, although the exact molecular mechanism remains unknown [174, 179].

Thus, both vacuolating and cell death-inducing effects depend on VacA functioning as an ion channel. At the same time, the available data indicate that vacuolization has no critical effect on VacA-induced cell death.

#### Epsilon toxin of Clostridium perfringens

Epsilon toxin (ET) is another well-known PFT inducing vacuolization of endosomal-lysosomal components. It is one of the main virulence factors of *Clostridium perfringens*, a bacterial infectious agent inducing anaerobic enterotoxemia in animals (Table 1). The mechanism of ET-induced vacuolization was studied in the MDCK cell model. ET was shown to bind to lipid rafts in membranes of the cell to form heptameric ion channels with slight selectivity for anions [25, 201]. Then the toxin is internalized into early endosomes, transported to LAMP-2-positive endosomal-lysosomal organelles, and induces their vacuolization. The vacuolating action of ET entirely relies on its functioning as an ion channel. As with VacA, the formation of ET-induced vacuoles is suppressed by inhibitors of chloride-selective channels and V-ATPase as well as by expression of dominant-negative GTPases Rab5 and Rab7 [25, 201].

ET-induced cell death was studied in the model of murine renal cortical collecting duct mpkCCDc14 cells. ET was shown to form ion channels in the cell membrane whose functioning sharply decreases the concentration of intracellular K^+^ and simultaneously increases the concentrations of Cl^−^, Na^+^, and Ca^2+^. Apart from modified ionic balance, the cytoplasm shows rapid decrease in the ATP level, which is followed by increased expression of Bax protein, induction of mitochondrial membrane permeability, cytochrome c release, and mitochondrial-nuclear translocation of AIF. At the same time, ET-induced cell death is not accompanied by the activation of executioner caspases and is not suppressed by caspases inhibitors. The morphological and biochemical properties of ET-induced cell death are similar to those of oncosis [202].

It is important to note that the cytotoxic effect of ET on mpkCCDc14 cells involves no toxin internalization and vacuole formation unlike that on MDCK cells [202]. These data indicate that vacuolization is not required for the cytotoxic effect of ET.

#### Vibrio cholerae сytolysin

Сytolysin (VCC), а virulence factor of *V. cholerae*, forms ion channels in the plasma membrane of intestinal epithelial cells and induces K^+^ release from the cytoplasm to the extracellular space. High VCC concentrations induce ionic imbalance in the cytoplasm, rapid depletion of the ATP pool, and cell death [203]. The drop in the ATP level seem to be due to the activation of ATP-dependent ion pumps similar to that observed in PLCD after opening of BKCa. Low VCC concentrations triggers autophagy; it is localized to autolysosomes and induces their vacuolization [143, 204–207] (Table 1). In contrast to VacA, VCC-induced vacuolization does not depend on the GTPase Rab7 and V-ATPase [205]. At the same time, VCC can induce vacuolization only in autophagy-competent cells. Autophagy inhibitors completely block vacuole formation [206]. Similar to VacA, VCC-induced vacuolization is not the cause of cell death. Conversely, inhibition of autophagy not only suppressed vacuolization but also enhanced the cytotoxic effect of VCC. Arguably, autophagy induction by VCC allows the cell to isolate a fraction of toxin molecules in autolysosomes to mitigate its impact on the cell [206].

#### Lysinibacillus sphaericus binary toxin

The binary toxin (Bin) from the bacterium *Lysinibacillus sphaericus*, which is used as a biopesticide against *Culex* and *Anopheles* mosquitoes [208], induces cell death accompanied by vacuolization of autolysosomes [209] (Table 1). Bin is composed of two subunits, A and B, which are synthesized as precursors and form parasporal crystal inclusions. These crystals dissolve when the bacterium appears in the mosquito larval midgut. Both subunits are processed by proteolytic enzymes of the gastric juice to form an active dimer. The dimer binds to the α-glucosidase Cpm1 (in *Culex* larvae [210]) or maltase Agm3 (in *Anopheles* larvae [211]) on the surface of midgut epithelial cells and forms nonselective ion channels in their plasma membrane. Experiments on MDCK cells expressing the toxin receptor Cpm1 demonstrated that, after localization to the cell membrane, the toxin is internalized, transported into recycling early endosomes, and comes back to the plasma membrane in them. Thus, the toxin does not enter acidic endosomal-lysosomal organelles where it could be degraded. In addition, the toxin is not found in the membranes of vacuolated autolysosomes. The mechanism by which Bin induces vacuolization of autolysosomes without being localized to them currently remains unknown [144].

An interesting feature of Bin-induced vacuolization of MDCK cells is its periodicity. Emerged vacuoles disappear in a short period of time and then reappear after cell division. The periodicity of vacuole appearance depends on autophagy activity changing in the cell cycle. Vacuoles disappear after autophagy activation observed some time after Bin enters the cell [144, 212, 213]. Autophagy activity reduces when toxin-containing cells divide [214], which in turn leads to the emergence of the secondary post-mitotic vacuoles. When autophagy activity is restored in divided cells, vacuoles are reduced again. Thus, autolysosome vacuolization depends inversely on autophagy activity [144].

The mechanism of Bin-induced cell death currently remains unknown. The functioning of ion channels formed by the toxin, which affects cytoplasmic ionic balance and induces cellular osmotic stress, was proposed as the cause of cell death [215–218]. The cytotoxic effect of Bin is cell type-dependent. For instance, Bin induces death of midgut epithelial cells in mosquito larvae but not of model MDCK cells. At the same time, the vacuolating effect of Bin is observed both in midgut epithelial and MDCK cells. Thus, vacuolization is clearly unrelated to the cytotoxic effect of Bin.

#### Other PFTs

Several other PFTs (aerolysin from *Aeromonas hydrophila*, αX toxin from *Xenorhabdus nematophila*, and hemolysin from *Serratia marcescens*) forming ion channels in the plasma membrane can induce vacuolization of ER components [145, 146]. The mechanism underlying their vacuolating effect remains unclear. Admittedly, the activity of toxin-formed channels leads to cellular osmotic stress, which activates p38 MAP kinase controlling the triggering of several defense responses including the unfolded protein response; and its long-term induction can trigger vacuolization of ER components [15, 70, 84, 219]. To date, no data are available on the relationship between the cytotoxic effect of these toxins and their vacuolating activity.

In conclusion of the discussion of bacterial PFTs, it can be stated that they can induce vacuolization of different organelles: endosomes, lysosomes, autolysosomes, and ER components. In all documented cases, vacuolization depends on the functioning of toxins as ion channels. At the same time, the cytotoxic effect of PFTs relies on mitochondrial dysfunction or cellular osmotic stress. Thus, vacuolization seem to be a byproduct of PFT functioning and has no effect on the cell death process.

## VIRAL INFECTIONS

The first reports on the capacity of viruses to induce cytoplasmic vacuolization in infected cells we are aware of date back to the middle of last century [220–222]. To date, the vacuolating effect has been demonstrated for many viral families. Among RNA viruses, these include picornoviruses (hepatitis A virus [223] and encephalomyocarditis virus [224, 225]), flaviviruses (hepatitis C virus [226–228], bovine virus diarrhea [229], dengue virus [230] and West Nile virus [231]), rhabdoviruses (rabies virus [232]), paramyxoviruses (Newcastle disease virus [233–235]), nodaviruses (viral nervous necrosis virus [236–239]), and retroviruses (murine leukemia virus [240], Rous sarcoma virus [241, 242], bovine leukemia virus [243], human and primate immunodeficiency viruses [244, 245]). DNA viruses inducing vacuolization include hepadnaviruses (hepatitis B virus [26]), polyomaviruses (SV40 [222, 246]), iridoviruses [247], and papillomaviruses [248].

Vacuolization of virus-infected cell is usually an indication of its imminent death (reviewed in [4, 249, 250]). In some cases, vacuolization is observed in infected cells undergoing necroptosis or paraptosis-like death [247, 251–256]. Factors of vacuolization for these cell death types were described above. At the same time, the mechanisms triggering these virus-induced cell death types remain underexplored. In addition, viruses can induce vacuolization not associated with necroptosis and PLCD. Such vacuolization is triggered by individual viral proteins, usually, envelope or capsid proteins. To date, the vacuolating effect was demonstrated for proteins of murine leukemia virus, SV40, human papillomavirus, and hepatitis A, B, and C viruses. The mechanisms underlying the vacuolating effect of these proteins are different.

### Murine leukemia virus

Neuropathogenic strains of murine leukemia virus (MuLV) induce *in vivo* cytoplasmic vacuolization and death of central nervous system cells: neurons, astrocytes, oligodendrocytes, and microglial cells [240, 257]. The vacuolating agent of neuropathogenic MuLV is the envelope protein (Env). Env is synthesized as a precursor with an N-terminal signal peptide for sorting to the ER and a domain responsible for binding to the viral receptor, CAT-1 transporter. After translocation to the ER, Env of nonpathogenic MuLV strains is subjected to processing and glycosylation, after which it leaves the ER. In the case of neuropathogenic MuLV strains, the amino acid sequences of the Env signal peptide and terminal region of the CAT-1-binding domain are modified [258]. As a result, Env is misfolded and accumulated in the ER. Misfolded Env interacts with the ER chaperone BiP, one of the main sensors of unfolded proteins in the ER. Altogether, this leads to ER stress and activation of the unfolded protein response [259, 260]. Apparently, long-term ER stress results in the accumulation of ROS, which interfere with correct protein folding, induce ERAD dysfunction, activate autophagy, and affect homeostasis in endosomal-lysosomal organelles. Ultimately, these events trigger cell death programs [257, 261–267]. As with paraptosis-like cell death, Env-induced vacuoles are formed from ER components due to the accumulation of the misfolded protein in the ER coupled with ERAD dysfunction. In addition, vacuoles are partially formed from fused endosomal-lysosomal organelles and autophagosomes [268].

The role of vacuolization in MuLV-induced cell death remains obscure. By analogy with paraptosis-like cell death, one can propose that vacuolization can contribute to recovery from Env-induced ER stress, and thus plays a pro-survival role.

### Hepatitis B virus

Hepatitis B virus (HBV) is a member of the hepadnavirus family. Apart from hepatitis, it causes malignant transformation in the liver and stomach [269]. The cytotoxic effect of HBV includes ER vacuolization induced by the large HBV surface antigen (L-HBsAg) [26, 270]. The vacuolating effect of L-HBsAg is due to the formation of aggregates and filament structures from it in the ER where it is localized. Aggregated forms of L-HBsAg are transported via the ER-Golgi intermediate compartment (ERGIC). The rate of L-HBsAg translocation across ERGIC is substantially slower than the rate of L-HBsAg translocation into the ER. The accumulation of aggregated L-HBsAg in the ERGIC and ER causes ERGIC dysfunction, impairs secretory function of the ER, and induces vacuolization [271]. L-HBsAg-induced disturbances seem to be the cause of cell death [26, 271–273]. At the same time, vacuolization increases the ER capacity, which allows the cell to cope with L-HBsAg aggregates accumulating in the ER for some time.

In addition to L-HBsAg, HBV envelope includes medium and small HBV surface antigens (M-HBsAg and S-HBsAg), which are also localized to the ER and secreted via ERGIC but have no vacuolating effect. All three proteins are translated from alternative start codons of the same gene and differ by the N-terminal domains. S-HBsAg and M-HBsAg promptly pass through the ERGIC and form pseudovirus particles, which are secreted to the extracellular space [271]. These data suggest that the N-terminal region absent in S-HBsAg and M-HBsAg is the determinant of the vacuolating and cytotoxic effects of L-HBsAg.

### Hepatitis C virus and other flaviviruses

Hepatitis C virus (HCV) of the flavivirus family induces lipid and nonlipid vacuolization of infected cells. Lipid vacuolization is observed in hepatocytes infected by HCV and results from the fusion of a large number of lipid droplets (mass lipid vacuolization in hepatocytes *in vivo* is called steatiosis [274]). Lipid droplets play an important role in the HCV cycle being a platform for the virus replication and assembly; and their abundance increases HCV reproduction rate [274, 275]. Vacuolization of lipid droplets is accomplished due HCV core protein (CP). CP forms a complex with diacylglycerol O-acyltransferase 1 (DGAT1), which is transported to lipid droplets and is localized to their surface. As a result, lipid droplets become inaccessible for cell lipases. In addition, CP regulates the expression of protein factors of lipid metabolism [276–279]. Integral action of CP increases the number of lipid droplets and induces their fusion yielding lipid vacuoles [274]. Thus, vacuolization of lipid droplets improves the efficiency of virus assembly but causes no cell death.

Nonlipid vacuolization is observed in HCV-infected hepatocellular carcinoma and lymphoma cells *in vitro* [226, 228, 280]. The relationship between nonlipid vacuolization and macroautophagy has been demonstrated in hepatocellular carcinoma HuH7 cells [228]. HCV infection of macroautophagy-deficient HuH7 cells induces vacuolization and cell death, while infected macroautophagy-competent HuH7 cells demonstrate autophagy induction without vacuolization and death. Thus, vacuolization is associated with the cytotoxic effect of HCV [228]. The origin of vacuoles in HuH7 cells has not been established. The relationship between vacuolization and autophagy activity suggests that vacuoles are formed from ER components under the influence of the viral CP. HCV CP was shown to enter the ER and induce its stress. Long-term stress leads to the ER dysfunction and cell death [281]. Since autophagy can prevent ER stress [282], it is possible that it also blocks ER vacuolization by participating in the degradation of HCV CP.

Several other flaviviruses have vacuolating effect. For instance, bovine viral diarrhea virus induces vacuolization of acidic endosomal-lysosomal organelles in infected cells [229]. Dengue and West Nile viruses induce the vacuole formation presumably from the ER components [231, 283, 284]. The mechanisms underlying the vacuolating effect of these viruses remain unknown.

### SV40

Simian virus 40 (SV40), a member of the polyomavirus family, can infect simian and human cells. Published data indicate that SV40 can be involved in renal diseases and has moderate oncogenic potential [285–288]. SV40-infected cells demonstrate two types of vacuolization, transient (primary) and irreversible (secondary). Transient vacuolization is observed at the initial infection stage before SV40-specific cytopathic changes appear. Irreversible cytopathic vacuolization results from virus multiplication and precedes infected cell death [289, 290]. The emergence of primary and secondary vacuoles follows swelling and clustering of acidic and endosomal-lysosomal organelles. Apparently, primary and secondary vacuoles have the same origin from fused endosomal-lysosomal organelles. At the same time, their content is not acidic and shows no activity of lysosomal enzymes [291].

The involvement of SV40 capsid protein VP1 in vacuolization process has been reported. This protein mediates virus entry via binding to ganglioside GM1, which resides on the plasma membrane surface and serves as the virus receptor. SV40 strains were obtained with VP1 mutations that affected binding to GM1. Such strains remain infectious (apparently, due to the utilization of another ganglioside receptor); however, they either induce no vacuolization or their vacuolating effect is substantially reduced [292–294]. Thus, the interaction between VP1 and GM1 seem to be the factor of SV40 vacuolating effect. However, the details of the vacuolization mechanisms currently remain obscure. Likewise, there is no data supporting that vacuolization is required for cell death.

### Hepatitis A virus

Hepatitis A virus (HAV) is the only representative of the picornavirus genus *Hepatovirus*. It causes acute hepatitis A, which can be followed by chronic form of the disease. Cytoplasmic vacuolization of hepatocytes and ductal epithelial cells is a common finding in acute hepatitis A [295, 296]. Vacuolization as well as other cytopathic changes such as multilayer membrane structures and tubular-vesicular network are observed in HAV-infected cells *in vitro* [223, 297]. Some of these morphological modifications, but not cytoplasmic vacuolization, are reproduced in cells infected by noncytopathogenic HAV strains as well as after individual expression of viral proteins 2B, 2C, and 2BC [223, 298]. The vacuole origin and mechanisms of formation currently remain unknown. At the same time, vacuoles similar to those found in HAV infection are observed upon expression of viral protease 3C (HAV3C) *in vitro* in A549 and Calu-1 cell lines. These vacuoles were shown to form by atypical fusion of several types of endosomal-lysosomal organelles [30] (Table 1). The vacuolization depended on proteolytic activity of HAV3C and did not develop upon expression of catalytically inactive variant of the protease. Hence, HAV3C can be the factor of cell vacuolization in HAV infection. Noteworthy, it is not an envelope or capsid protein that has a vacuolating effect in HAV infection unlike virus-induced vacuolization cases described above. In addition to vacuolization, HAV3C can induce caspase-independent cell death. However, the cytotoxic and vacuolating effect of HAV3C are not mutually related since bafilomycin A1, an inhibitor of V-ATPase, completely blocks vacuolization but has no effect on the protease cytotoxic effect [30]. The data obtained on HAV3C suggest that cytoplasmic vacuolization is not related to cell death in HAV infection.

### Human papillomavirus

Human papillomavirus (HPV) is one of the major causes of anogenital malignancies as well as head and neck cancers [299, 300]. HPV induces the formation of multiple perinuclear vacuoles in infected epithelial cells and keratinocytes. Their fusion gives rise to one or several large vacuoles that occupy nearly the whole cell. As a result, HPV-infected cells look as though they have no content. The typical morphology is the origin of their name, koilocytes or “empty cells.” The significance of vacuolization for HPV is not clear since its life cycle occurs in the nucleus. Koilocytes are thought to become fragile and lyse, which releases virions from the nucleus and favors virus spread [248]. Thus, the available data suggest that vacuole formation is the direct cause of HPV-infected cell death.

Vacuolization observed in HPV-infected cells can be reproduced *in vitro* by co-expression of viral proteins E5 and E6 [248]. This is another example (in addition to HAV 3C protease) of non-envelope or non-capsid viral proteins with a vacuolating effect. The mechanism of E5- and E6-induced vacuolization as well as the origin of vacuoles remain unknown. At the same time, E5 was reported to be associated with the nuclear and ER membranes, and its C-terminal region interacts with calpactin-1 and karyopherin β-3 proteins involved in the regulation of vesicular transport into the nucleus and endosomal-lysosomal organelles. These interactions result in perinuclear localization of calpactin-1 and karyopherin β-3, which can contribute to vacuole formation [301–304].

To summarize the above data on vacuolating effect of viruses, vacuolization is due to individual proteins largely composing the envelope or capsid in most studied cases. At the same time, different proteins induce vacuolization by different mechanisms. Generally, vacuolization results from dysfunction of the ER or endosomal-lysosomal organelles. Vacuolating proteins of a number of viruses (MuLV, HBV, and HPV) induce cell death by reproducing the cytotoxic effect of the whole virus. Vacuolization in these cases results from ER dysfunction, which is the direct cause of cell death. At the same time, the proper vacuolization either has no effect on cell death or conversely protects the cell by increasing the ER capacity and by reducing stress.

## CONCLUSIONS

Compilation of current data on molecular mechanisms of vacuole formation in mammalian cells induced by chemical, bacterial, and viral agents as well as the relationship of vacuolization to cell death and survival indicates that in most cases cytoplasmic vacuoles are formed from components of the endoplasmic reticulum or endosomal-lysosomal organelles. At the same time, vacuolization of components of the same compartment can rely on different mechanisms in an inducer-dependent manner.

ER vacuolization can be triggered by cellular osmotic stress (e.g., after hyperactivation or inhibition of BKCa channels or exposure to PFT) or by accumulation of excessive protein in the ER. In cellular osmotic stress, ER vacuolization proceeds due to mitochondrial dysfunction and ATP pool depletion, which are causes of cell death. In these cases, vacuole formation appears to have no significant effect on cell death process. ER overload leads to ERAD dysfunction and blocks secretory activity of the ER and/or ERGIC. These abnormalities are direct causes of cell death. However, vacuolization induced by these events can have a pro-survival effect due to the increased ER capacity and decreased protein content in ER, although this has not been directly confirmed by experiments.

Vacuolization of endosomal-lysosomal components is related to disturbed sorting and/or fusion of the organelles or changed intraorganellar ionic balance, which lead to the dysfunction of macropinocytosis, endocytosis, and autophagy. Vacuolization of endosomal-lysosomal organelles is not the cause of cell death in most cases (e.g., after the exposure to some methuosis inducers or toxins VacA, Bin, SubAB, and Stx2). Apparently, the emergence of koilocytes in HPV infection is the only exception. In some cases, the membranes of vacuolated endosomal-lysosomal organelles accumulate bacterial toxins (e.g., VCC and VacA), which can decrease their harmful influence and exert a pro-survival effect.

It should be noted that despite the large volume of data on vacuolization inducers, the mechanisms underlying their action remain unclear for many bacterial toxins, viruses, and low-molecular weight inducers of cell death accompanied by vacuolization. Therefore, in most cases the effect of vacuolization on cell death and survival can hardly be evaluated. At the same time, the data analyzed, as well as general conclusion that the formation of vacuoles is not the cause of cell death, allow to consider vacuolization as a side effect of the action of cytotoxic factors. However, given the incomplete data about the mechanisms of vacuolization, it remains possible that, in at least some cases, vacuole accumulation is an important initiating event, causing metabolic alterations or stress responses that lead to cell death, albeit indirectly.

In summary, there are not many cases when cytoplasmic vacuole formation directly causes cell death. Commonly, vacuolization has no effect on cell death or even can reduce stress and increase the cell survival potential. At the same time, there is no data indicating that vacuolization is a programmed cell response aimed at modulating pro-death or pro-survival effects.

## OUTLOOKS

Despite the absence of direct relationship between vacuolization and cell death, the origin and properties of vacuoles can be indicative of the cell death type. Vacuolization of ER is typical of paraptosis and paraptosis-like cell death. Simultaneous vacuolization of ER and Golgi components are indicative of necroptosis or oncosis. Vacuolization of macropinosomes takes place in methuosis, while vacuolization of acidic endosomal-lysosomal organelles accompanies autophagy-associated cell death.

Thus, the identification of vacuole origin and properties can be helpful in elucidating the mechanisms of pathomorphological effect of vacuolization inducers. One can expect that ER vacuolization inducers can suppress protein folding, affect the ERGIC and ERAD systems, or alter ionic balance in the ER through cellular osmotic stress or dysregulation of channels in the ER membranes. At the same time, inducers of vacuolization of endosomal-lysosomal organelles can have a passive (mediated by the action of weak bases) or active (mediated by the formation of ion channels in organellar membranes) osmotic effect, stimulate hyperactivation of different endocytosis types, or interfere with normal functioning of organelles to stimulate their uncontrolled fusion or abnormal maturation. Consequently, vacuolization mechanisms will be studied primarily in the context of cell death although vacuolization does not cause cell death in most cases.

Discussing the importance of vacuolization for the study of known and not yet discovered types of cell death, it should be noted that cytoplasmic vacuolization accompanies programmed death of not only mammalian cells (which is the main subject of this review), but also of other organisms (e.g., roundworms, insects and protozoa). This allowed to put forward an intriguing hypothesis that cytoplasmic vacuolization is the phenotypic manifestation of an evolutionary conservative mechanism of cell death, that appeared in unicellular and preserved in multicellular organisms [305]. Indeed, contenders for the role of this evolutionary conservative mechanism could be PLCD and autophagic cell death, that are observed in the cells of Protists (e.g., slime mold *Dictyostelium discoideum* and some dinoflagellates), insects and mammals [60, 306, 307]. However, the term PLCD encompasses several types of cell death, causing vacuolization ER by different mechanisms (as it was reviewed above). As for autophagy, its activation accompanies some types of mammalian cell death, however in most cases autophagy does not drive it (reviewed in [307–310]). In those few examples when autophagy is responsible for cell death, the accumulation of a large number of autophagic organelles, but not formation of cytoplasmic vacuoles, occurs. In contrast, when cell death is accompanied by vacuolization of autophagic organelles, the reason of cell death is autophagy impairment, but not its activation [28, 134].

Thus, neither PLCD, nor autophagy-associated cell death represent the evolutionary conservative mechanism, which causes vacuolized phenotype. At the same time, the vacuolization itself is a conservative evolutionary morphological phenomenon, but is not a consequence of the execution of any specific conservative cell death program.
